# Investigating the phylogenetic placement of ectoparasitic families Cyclotornidae and Epipyropidae: Multigene insights into Zygaenoidea systematics (Lepidoptera)

**DOI:** 10.3897/zookeys.1291.204651

**Published:** 2026-09-14

**Authors:** Yanpeng Cai, Aihui Yin

**Affiliations:** 1 Molecular Diagnostic Research Center, Guizhou University of Traditional Chinese Medicine, Guiyang 550025, Guizhou, China Molecular Diagnostic Research Center, Guizhou University of Traditional Chinese Medicine Guiyang China

**Keywords:** Cyclotornidae, Epipyropidae, mitogenome, phylogeny, Zygaenoidea

## Abstract

Zygaenoidea is a highly diverse lepidopteran superfamily whose monophyly remains unresolved, particularly regarding the phylogenetic positions of the two ectoparasitic families, Cyclotornidae and Epipyropidae. In this study, multi-gene datasets comprising 22 genes (13 mitochondrial protein-coding genes (PCGs), 2 mitochondrial rRNA genes, and 7 nuclear PCG fragments) were assembled from 62 representative samples covering all 11 recognized zygaenoid families and all 29 ditrysian superfamilies. Maximum likelihood and Bayesian inference methods were used to reconstruct phylogenetic relationships. Our results consistently showed that the traditionally defined Zygaenoidea was non-monophyletic. Cyclotornidae and Epipyropidae were recovered together in most of our analyses, often with moderate to strong support, and were placed near the base of Apoditrysia, distantly from the core Zygaenoidea, possibly as sister to Douglasioidea. However, given the long branches leading to these two lineages, this sister-group relationship should be interpreted with caution pending further evidence. In contrast, the remaining nine families constituted a well-supported monophyletic group (core Zygaenoidea). Based on these findings, we propose that Zygaenoidea*sensu stricto* should include only the nine core families and exclude Cyclotornidae and Epipyropidae. This study provides a robust phylogenetic framework for Zygaenoidea and offers new insights into the higher-level systematics of Ditrysia.

## Introduction

Zygaenoidea represents one of the most species-rich superfamilies of Lepidoptera, with approximately 3550 described species worldwide (van Nieukerken et al. 2011; Matson et al. 2019; Lourido 2021; Mirić et al. 2024; Epstein et al. 2025), and reaches its highest diversity in tropical and subtropical regions. Eleven extant families are currently recognized within the superfamily: Aididae (7 spp.), Cyclotornidae (5 spp.), Epipyropidae (32 spp.), Heterogynidae (10 spp.), Himantopteridae (ca. 80 spp.), Lacturidae (ca. 140 spp.), Limacodidae (ca. 1880 spp.), Megalopygidae (ca. 230 spp.), Phaudidae (15 spp.), Somabrachyidae (8 spp.), and Zygaenidae (ca. 1142 spp.). Among these, Limacodidae and Zygaenidae are by far the largest and most diverse lineages, together accounting for approximately 85% of all described species in Zygaenoidea. Most of the remaining smaller families (excluding Cyclotornidae and Epipyropidae) have been inferred to be closely related to either Limacodidae or Zygaenidae (Epstein 1996; Epstein et al. 1998).

Zygaenoidea encompasses a diverse assemblage of ditrysian moths exhibiting extreme ecological and morphological specialization. The superfamily includes well-known lineages such as chemically defended burnet moths (Zygaenidae, Zygaeninae) (Mirić et al. 2024), slug caterpillars (Limacodidae) (Epstein et al. 1998), and venomous flannel moths (Megalopygidae) (Dellinger and Day 2020). Some members of this superfamily exhibit a unique ability to biosynthesize or sequester cyanogenic glucosides for chemical defense (Davis and Nahrstedt 1987; Epstein et al. 1998). Adults of Zygaeninae actively exude hydrogen cyanide droplets from specialized cephalic cavities when disturbed (Jones et al. 1962; Niehuis et al. 2006). This toxicity is advertised by striking aposematic coloration (typically metallic black with red or yellow markings) linked to diurnal activity (Mirić et al. 2024).

Zygaenoid larvae are predominantly exposed external folivores (Mitter et al. 2017). In marked contrast, the two small and enigmatic families Cyclotornidae and Epipyropidae represent the only lepidopteran lineages whose larvae have evolved an obligate ectoparasitic life history (Goldstein 2017). First instars of both groups employ piercing mandibles to attack and cling to auchenorrhynchan hemipteran hosts (Efetov and Tarmann 2017). Epipyropidae exhibits a cosmopolitan distribution (excluding Antarctica), with larvae ectoparasitizing cicadas (Cicadidae) as well as plant- and leafhoppers (Cicadellidae, Delphacidae, and Fulgoridae) (Liu et al. 2018; Meyer-Rochow et al. 2023). Cyclotornidae, endemic to Australia, displays a more complex life history: larvae initially develop as external parasites of leafhoppers and scale insects, but subsequently abandon their hemipteran hosts to become obligate predators of ant brood within nests (Pierce and Dankowicz 2022; Meyer-Rochow et al. 2023).

No phylogenetic study has yet been specifically designed to test the monophyly of Zygaenoidea as a whole. Although several family-level phylogenetic investigations have addressed relationships within Limacodidae or Zygaenidae, as well as among their allied families (Niehuis et al. 2006; Zaspel et al. 2016; Lin et al. 2019; Liang et al. 2024; Mirić et al. 2024; Epstein et al. 2025), none of these studies have included all 11 currently recognized families of the superfamily. Notably, Cyclotornidae and Epipyropidae have not been included in any of these investigations. Despite this sampling gap, a consistent pattern has emerged: the remaining nine families reliably form a well-supported monophyletic group, referred to as the ‘core Zygaenoidea’.

Cyclotornidae and Epipyropidae have only been sampled in comprehensive phylogenetic studies covering the entirety of Lepidoptera. In these broader analyses, their inferred phylogenetic positions within Ditrysia have been highly unstable, varying markedly across studies. Both lineages typically exhibit long branches, and crucially, they almost never cluster with the core Zygaenoidea (Mitter et al. 2017); moreover, they do not even consistently form a sister-group relationship with each other (e.g., Mutanen et al. 2010; Cho et al. 2011; Regier et al. 2013; Heikkilä et al. 2015). In several studies, Cyclotornidae and/or Epipyropidae were sometimes recovered within Cossoidea, a superfamily considered arguably to be the closest relative of Zygaenoidea, but such placements invariably received weak nodal support (e.g., Regier et al. 2009; Cho et al. 2011; Bazinet et al. 2013; Regier et al. 2013; Rota et al. 2022). In the most recent phylogenomic investigation to include Epipyropidae, the family clustered with neither the core Zygaenoidea nor Cossoidea, but instead was placed as sister to Schreckensteinioidea (Chen et al. 2025).

Despite extensive phylogenetic work on Lepidoptera, the monophyly of Zygaenoidea and the placement of its two ectoparasitic families, Cyclotornidae and Epipyropidae remain unresolved. No study has specifically targeted all 11 recognized zygaenoid families. To address this gap, we assembled phylogenetic datasets comprising 22 genes (13 mitochondrial protein-coding genes (PCGs), two mitochondrial rRNA genes, and seven nuclear PCG fragments) across 62 representative samples, covering all Zygaenoidea families and all 29 currently valid ditrysian superfamilies. Specifically, our superfamily-level objectives are to: (1) test the monophyly of Zygaenoidea*sensu lato*; (2) clarify the phylogenetic relationship between Cyclotornidae and Epipyropidae; (3) offer an updated taxonomic framework for the superfamily; and (4) offer further insights into the phylogenetic framework of Ditrysia.

## Materials and methods

### Gene sampling

A total of 22 genes were included in this study: 13 full-length mitochondrial PCGs, two full-length mitochondrial rRNA genes, and seven nuclear PCG fragments (Suppl. material 1). Mitochondrial genes were extracted from mitochondrial genomes available in GenBank. For species lacking a publicly available mitochondrial genome, we attempted to assemble one from the corresponding SRA files, when available, and then extracted the mitochondrial genes. Most nuclear gene fragments were obtained from GenBank, including carbamoyl phosphate synthetase domain protein (*CAD*), two fragments of elongation factor 1 alpha (*EF-1α*), glyceraldehyde-3-phosphate dehydrogenase (*GAPDH*), isocitrate dehydrogenase (*IDH*), cytosolic malate dehydrogenase (*MDH*), ribosomal protein S5 (*RpS5*), and *wingless*.

Lepidopteran mitochondrial genomes are abundant in GenBank, and the 13 mitochondrial PCGs along with the two rRNA genes have been widely used in recent phylogenetic studies of Lepidoptera (e.g., Wang et al. 2018; [Bibr B12][Bibr B33]). The seven nuclear gene fragments are the most commonly amplified markers in Lepidoptera and have been successfully employed in many previous molecular phylogenetic studies (e.g., Wahlberg and Wheat 2008; Heikkilä et al. 2015; Brown et al. 2019; Wang and Li 2020; Mirić et al. 2024). However, combining these genes together in a single phylogenetic study has remained rare.

### Taxon sampling

This study included a total of 75 lepidopteran species (Table 1, Suppl. material 1), merged into 62 final samples (Suppl. material 1). The ingroup comprised all 29 recognized ditrysian superfamilies plus two ditrysian families of uncertain placement (Heliocosmidae and Millieriidae), while three glossatan superfamilies served as outgroup (Suppl. material 1).

**Table 1.** List of all sampled species used for phylogenetic analyses, showing superfamilies, families and species names with authorities and publication years.

**Superfamily**	**Family**	**Species**
**Ingroup**		
Alucitoidea	Alucitidae	*Alucita hexadactyla* Linnaeus, 1758
Bombycoidea	Bombycidae	*Bombyx mori* Linnaeus, 1758
	Saturniidae	*Saturnia pavonia* Linnaeus, 1758
Calliduloidea	Callidulidae	*Pterodecta felderi* (Bremer, 1864)
	Whalleyanidae	*Whalleyana vroni* Viette, 1977
Carposinoidea	Carposinidae	*Carposina smaragdias* Turner, 1916
		*Carposina sasakii* Matsumura, 1900
Choreutoidea	Choreutidae	*Choreutis nemorana* (Hübner, 1799)
		*Prochoreutis myllerana* (Fabricius, 1794)
Cossoidea	Cossidae	*Cossus cossus* Linnaeus, 1758
		*Phragmataecia castaneae* (Hübner, 1790)
	Sesiidae	*Sesia apiformis* (Clerck, 1759)
Douglasioidea	Douglasiidae	*Tinagma ocnerostomellum* (Stainton, 1850)
		*Tinagma perdicella* (Zeller, 1839)
Drepanoidea	Doidae	*Doa* sp1
		*Doa* sp2
	Drepanidae	*Drepana curvatula* (Borkhausen, 1790)
Epermenioidea	Epermeniidae	*Epermenia illigerella* (Hübner, 1813)
		*Phaulernis dentella* (Zeller, 1839)
Galacticoidea	Galacticidae	*Homadaula lasiochroa* Lower, 1899
		*Homadaula anisocentra* Meyrick, 1922
Gelechioidea	Depressariidae	*Carcina quercana* (Fabricius, 1775)
	Gelechiidae	*Dichomeris juniperella* (Linnaeus, 1761)
	Oecophoridae	*Endrosis sarcitrella* Linnaeus, 1758
Geometroidea	Epicopeiidae	*Epicopeia hainesii* Holland, 1889
	Geometridae	*Alsophila aescularia* (Denis & Schiffermüller, 1775)
Gracillarioidea	Gracillariidae	*Gracillaria syringella* (Fabricius, 1794)
Hyblaeoidea	Hyblaeidae	*Hyblaea puera* (Cramer, 1777)
Immoidea	Immidae	*Imma loxoscia* Turner, 1913
		*Imma tetrascia* Lower, 1912
Lasiocampoidea	Lasiocampidae	*Lasiocampa quercus* Linnaeus, 1758
Mimallonoidea	Mimallonidae	*Mimallo amilia* (Cramer, 1780)
		*Lacosoma valva* (Schaus, 1905)
Noctuoidea	Noctuidae	*Noctua fimbriata* (Schreber, 1759)
	Nolidae	*Earias clorana* (Linnaeus, 1761)
Papilionoidea	Hesperiidae	*Thymelicus lineola* (Ochsenheimer, 1808)
	Papilionidae	*Papilio glaucus* Linnaeus, 1758
Pterophoroidea	Pterophoridae	*Stenoptilia veronicae* Karvonen, 1932
Pyraloidea	Crambidae	*Elophila nymphaeata* Linnaeus, 1758
	Pyralidae	*Pyralis farinalis* Linnaeus, 1758
Schreckensteinioidea	Schreckensteiniidae	*Schreckensteinia festaliella* (Hübner, 1919)
Simaethistoidea	Simaethistidae	*Metaprotus* sp.
Thyridoidea	Thyrididae	*Rhodoneura terminalis* (Walker, 1865)
		*Rhodoneura mellea* (Saalmüller, 1881)
		*Thyris fenestrella* (Scopoli, 1763)
Tineoidea	Tineidae	*Tinea pellionella* Linnaeus, 1758
Tortricoidea	Tortricidae	*Bactra lancealana* (Hübner, 1799)
		*Pandemis cinnamomeana* (Treitschke, 1830)
Urodoidea	Urodidae	*Urodus decens* Meyrick, 1925
		*Wockia asperipunctella* (Bruand, 1850)
Yponomeutoidea	Lyonetiidae	*Leucoptera malifoliella* (Costa, 1836)
Zygaenoidea	Aididae	*Aidos osorius* (Herrich-Schäffer, 1856)
	Cyclotornidae	*Cyclotorna ementita* Meyrick, 1921
		*Cyclotorna* sp.
	Epipyropidae	*Epipomponia nawai* Dyar, 1904
		*Fulgoraecia exigua* (Edwards, 1882)
		*Heteropsyche* sp1
		*Heteropsyche* sp2
	Heterogynidae	*Heterogynis penella* (Hübner, 1818)
		*Heterogynis canalensis* Chapman, 1904
	Himantopteridae	*Himantopterus fuscinervis* Wesmael, 1836
	Lacturidae	*Lactura subfervens* (Walker, 1854)
	Limacodidae	*Apoda limacodes* (Hufnagel, 1766)
	Megalopygidae	*Megalopyge arpi* (Schaus, 1900)
		*Megalopyge crispata* (Packard, 1864)
	Phaudidae	*Phauda mimica* Strand, 1915
		*Phauda flammans* (Walker, 1854)
	Somabrachyidae	*Somabrachys aegrota* (Klug, 1830)
	Zygaenidae	*Zygaena filipendulae* (Linnaeus, 1758)
incertae sedis	Heliocosmidae	*Heliocosma incongruana* (Walker, 1863)
		*Heliocosma* sp.
	Millieriidae	*Millieria dolosalis* (Heydenreich, 1851)
**Outgroup**		
Adeloidea	Incurvariidae	*Incurvaria pectinea* Haworth, 1828
Eriocranioidea	Eriocraniidae	*Eriocrania semipurpurella* (Stephens, 1835)
Tischerioidea	Tischeriidae	*Tischeria ekebladella* (Bjerkander, 1795)

To maximize gene coverage, we preferentially selected species with both mitogenome/SRA and nuclear data available from GenBank as superfamily representatives (44 final samples). Where congeneric species had complementary data (one with mitogenomic data only, the other with nuclear data only), we combined them into a single composite sample (12 final samples). Only one composite sample involved two confamilial species from different genera. We are confident that any species-level heterogeneity introduced by such combinations is unlikely to affect the superfamily-level phylogeny, which is the primary focus of this study.

Two additional samples were represented solely by nuclear genes, and one sample solely by mitogenome. For two critical samples, *Somabrachys aegrota* (Zygaenoidea, Somabrachyidae) and *Metaprotus* sp. (the only representative with DNA data available for the superfamily Simaethistoidea), we used three mitochondrial genes (*NAD1*, *16S rRNA*, *12S rRNA*) and a 657-bp *COX1* barcode sequence, respectively.

### Laboratory work

Three nuclear gene fragments (*EF-1α*, *IDH*, and *RpS5*) of *Epipomponia nawai*, a focal epipyropid species in this study were sequenced for the first time (Suppl. material 1). The specimen was a final-instar larva collected from the body surface of its cicada host *Trombonia linearis* (Walker, 1850), captured at Zhongpai Village (26°21'23"N, 106°53'40"E, 1605 m a.s.l.), Longli County, Qiannan Buyi and Miao Autonomous Prefecture, Guizhou, China, on 25 July 2025. Larval identification was based on external morphology following published taxonomic references (Jeon et al. 2002; Liu et al. 2018; Meyer-Rochow et al. 2023) and supported by DNA barcoding. The host cicada was identified via DNA barcoding.

Total genomic DNA was extracted from the larval body using the Rapid Animal Genomic DNA Isolation Kit (Sangon Biotech, Shanghai, China) following the manufacturer’s protocol. PCR amplification of the targeted genes was performed using the Direct PCR Kit (Sangon Biotech, Shanghai, China) with the primers listed in Suppl. material 2: table S2, following the manufacturer’s standard protocol. PCR products were sent to Sangon Biotech (Shanghai, China) for bidirectional Sanger sequencing.

For 16 representative species lacking public mitogenomes but with available WGS or transcriptomic SRA data, we attempted mitogenome assembly from these datasets (Suppl. material 1). Owing to variable SRA data quality, eight yielded complete or near-complete mitogenomes, while the remaining eight were only partially assembled with multiple gaps (Suppl. material 1).

Mitogenomes were assembled using multiple software packages as appropriate: ARC v. 1.1.3 (Hunter et al. 2015), GetOrganelle v. 1.7.7.1 (Jin et al. 2020), MitoZ v. 3.6 (Meng et al. 2019), NOVOPlasty v. 4.3.3 (Dierckxsens et al. 2017), and SPAdes v. 4.1.0 (Prjibelski et al. 2020). Gap filling and polishing of the assembled sequences were performed using Pilon v. 1.24 (Walker et al. 2014). Annotation was conducted with MitoZ and the MITOS2 online server (Galaxy v. 2.1.9) (Al Arab et al. 2017; Donath et al. 2019), followed by manual inspection and verification.

Mitogenes were extracted into individual FASTA files from annotated mitogenome sequences using a custom Python script.

The 20 protein-coding genes (7 nuclear PCG fragments and 13 mitochondrial PCGs) were aligned as codons using MACSE v. 2.07 (Ranwez et al. 2018), implemented in PhyloSuite v. 2 (Zhao et al. 2025). Poorly aligned regions were removed using Gblocks v. 0.91b (Talavera and Castresana 2007). The two non-protein-coding mitochondrial rRNA genes were aligned with MAFFT v. 7.52 (Katoh and Standley 2013) using the L-INS-i algorithm, and ambiguous positions were trimmed with trimAl v. 1.4.1 (Capella-Gutiérrez et al. 2009). Individual gene alignments were then concatenated in MEGA v. 11.0.13 (Tamura et al. 2021) to construct five datasets. The heterogeneity of sequence divergences was analyzed separately for each DNA dataset using AliGROOVE v. 1.0.8 (Kück et al. 2014) with default parameters.

### Datasets

The five datasets were as follows: (1) PCG123R: all three codon positions of the 20 PCGs plus two rRNA genes (17,507 sites); (2) PCG123: all three codon positions of the 20 PCGs (15,549 sites); (3) PCG12R: the first and second codon positions of PCGs plus two rRNA genes (12,324 sites); (4) PCG12: the first and second codon positions of PCGs (10,366 sites); (5) AA: amino acid sequences translated from the 20 PCGs (5,088 sites).

### Phylogenetic reconstructions

We reconstructed a total of 11 phylogenetic trees using Maximum Likelihood (ML) and Bayesian Inference (BI) methods. ML analyses were performed for all five datasets using RAxML-NG v. 2.0.0 (Kozlov et al. 2019), under partitioned substitution models selected by ModelFinder v. 2.2.2 (Kalyaanamoorthy et al. 2017) in PhyloSuite (Suppl. material 2: tables S3, S5, S7, S9, S11). Branch support was estimated from 1000 bootstrap (BS) replicates. BI analyses were conducted for all five datasets using MrBayes v. 3.2.7 (Ronquist et al. 2012) with partitioned substitution models selected by ModelFinder (Suppl. material 2: tables S4, S6, S8, S10, S12). Depending on the dataset, eight or 16 independent Markov chains were run for 11–35 million generations, sampling every 50–2000 generations. Convergence was assessed with Tracer v. 1.7.2 (Rambaut et al. 2018), ensuring effective sample sizes (ESS) > 200 and potential scale reduction factors (PSRF) approaching 1.0 (Gelman and Rubin 1992). The first 25–40% of trees were discarded as burn-in, and majority-rule consensus trees with nodal posterior probabilities (PP) were generated from the remaining trees.

For the AA dataset, an additional BI analysis was performed using PhyloBayes-MPI v. 1.8c (Lartillot et al. 2013) under the site-heterogeneous CAT-GTR model. Two independent chains were run for 80,000 generations until convergence (maxdiff < 0.3, ESS > 200). The first 30% of sampled trees were discarded as burn-in, and the remaining trees were used to construct the final consensus tree.

## Results and discussion

### Tests of heterogeneity of sequence divergences

**Figure 1.**
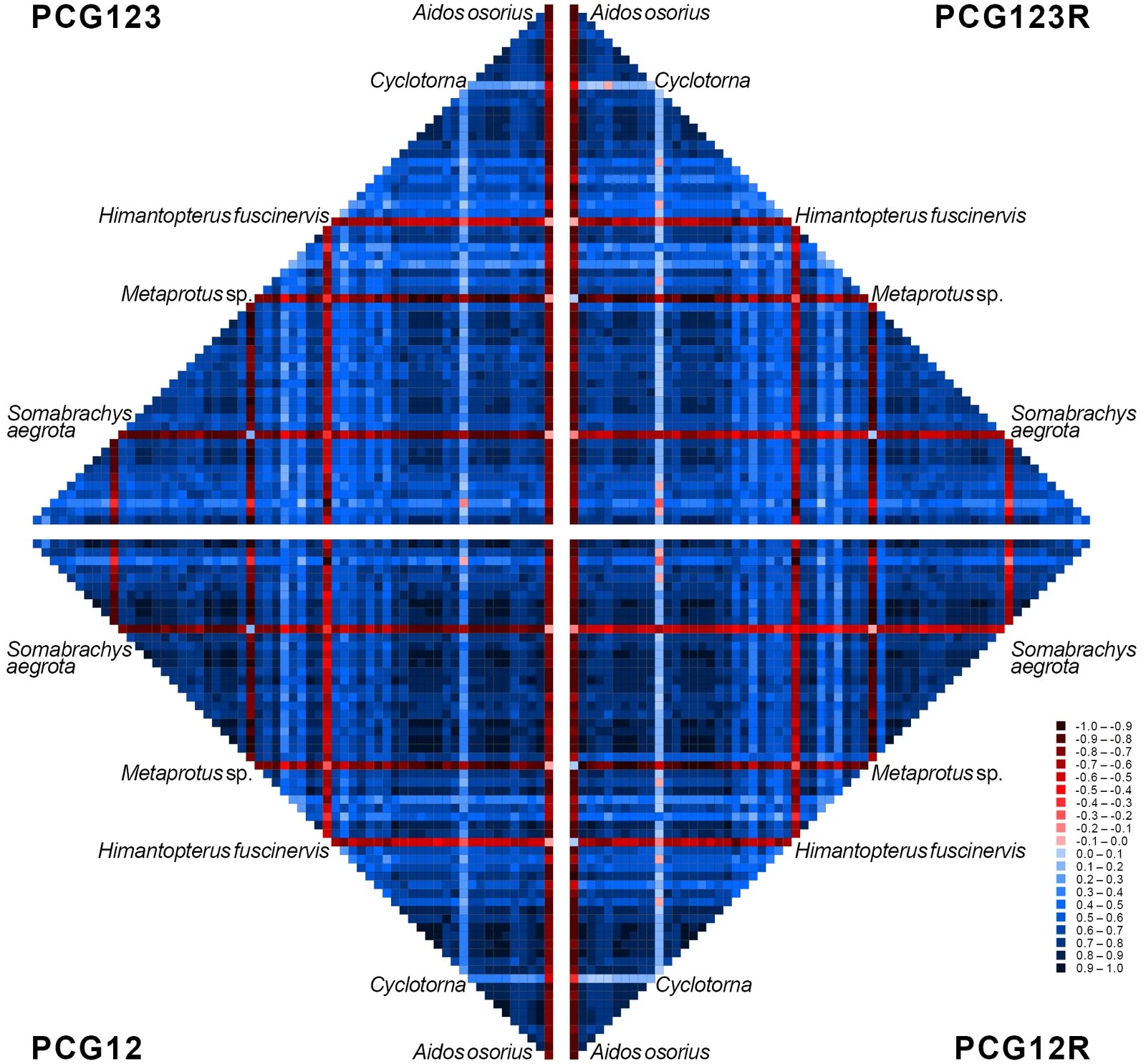
Heterogeneity of sequence divergences across the four DNA datasets. AliGROOVE scores are visualized as colored blocks, ranging from −1 (strong heterogeneity, red) to +1 (weak heterogeneity, blue). Sample labels: species names are given when nuclear and mitochondrial genes originate from the same species; genus name only when derived from different species of the same genus.

The AliGROOVE tests revealed generally low sequence divergence heterogeneity among the 62 representative samples across all four DNA datasets, with the exception of five samples: *Aidos osorius* (Zygaenoidea, Aididae; only nuclear genes available), *Himantopterus fuscinervis* (Zygaenoidea, Himantopteridae; only nuclear genes available), *Somabrachys aegrota* (Zygaenoidea, Somabrachyidae; only three mitogenes available), *Cyclotorna* (Cyclotornidae; mitogenome relatively poorly assembled), and *Metaprotus* sp. (Simaethistoidea, Simaethistidae; only the *COX1* barcode sequence available) (Fig. 1). The higher heterogeneity observed in these five samples was primarily due to missing genes or gene fragments.

### Phylogenetic analyses

**Figure 2.**
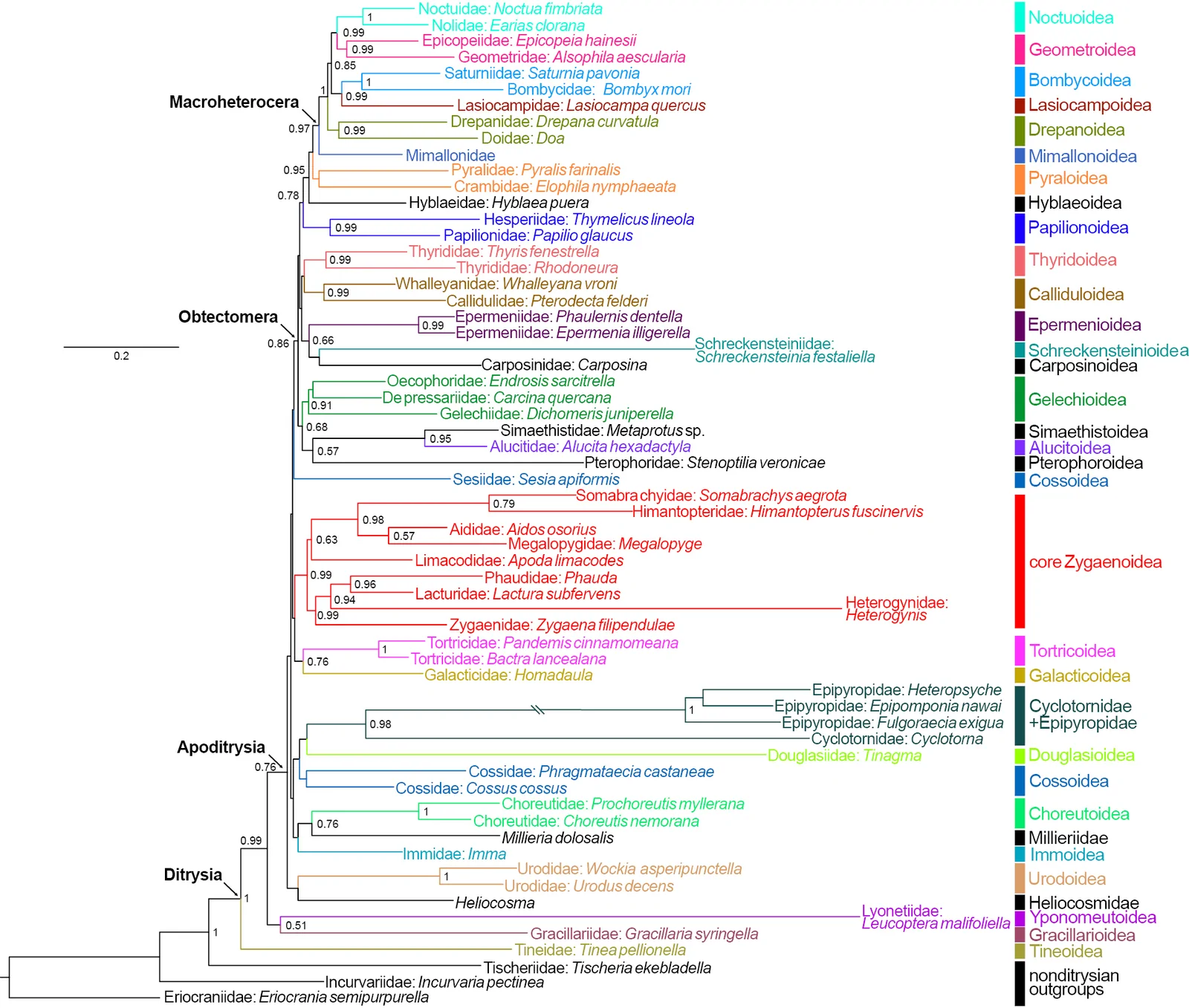
Bayesian inference phylogeny of Ditrysia (primary tree) based on the AA dataset under the CAT-GTR model. Node labels indicate Bayesian posterior probabilities (PP > 0.5 shown). Tip labels are formatted as follows: full family and species names when all genes originate from the same species; family and genus names when genes come from different species of the same genus; and family name only when genes are derived from different genera.

**Figure 3.**
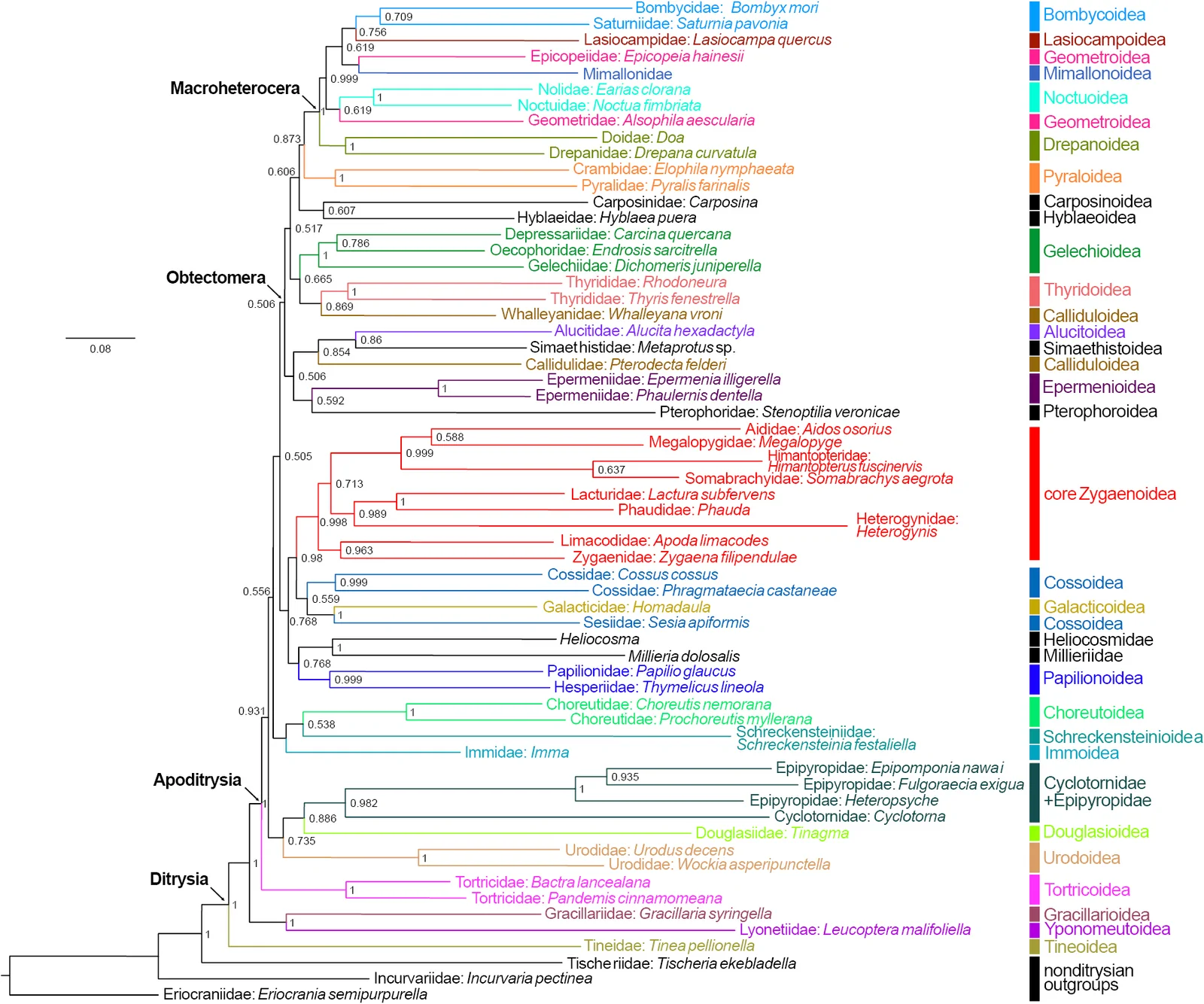
Bayesian inference (BI) tree constructed with MrBayes for PCG123 dataset. Node labels indicate Bayesian posterior probabilities (PP > 0.5 shown). Tip label format: full family and species names when all genes originate from the same species; family and genus names when genes come from different species of the same genus; family name only when genes are derived from different genera.

**Figure 4.**
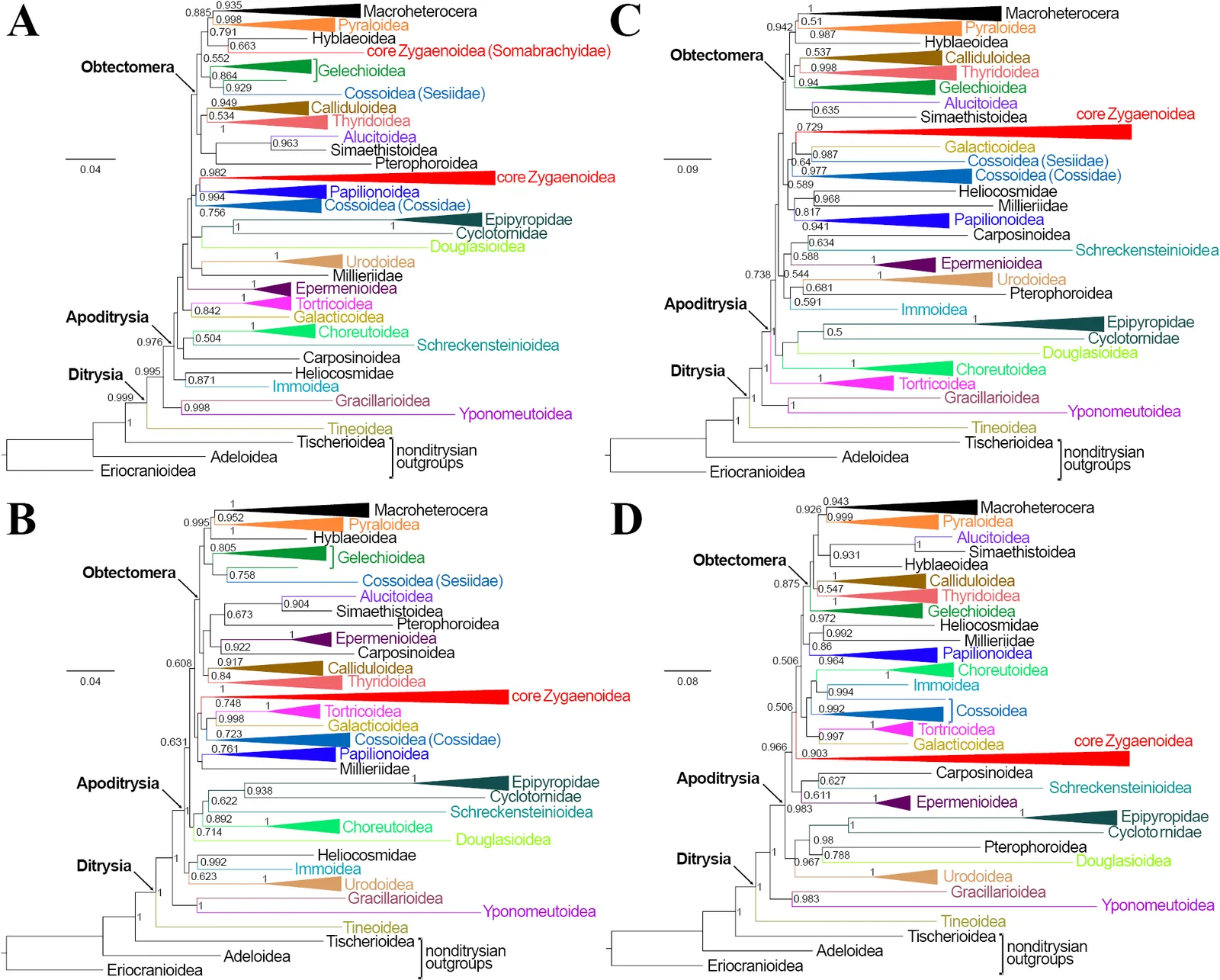
Partially collapsed Bayesian inference (BI) trees constructed with MrBayes. Node labels indicate Bayesian posterior probabilities (PP > 0.5 shown). **A–D**. BI trees for the PCG12, PCG12R, PCG123R, and AA datasets, respectively.

**Figure 5.**
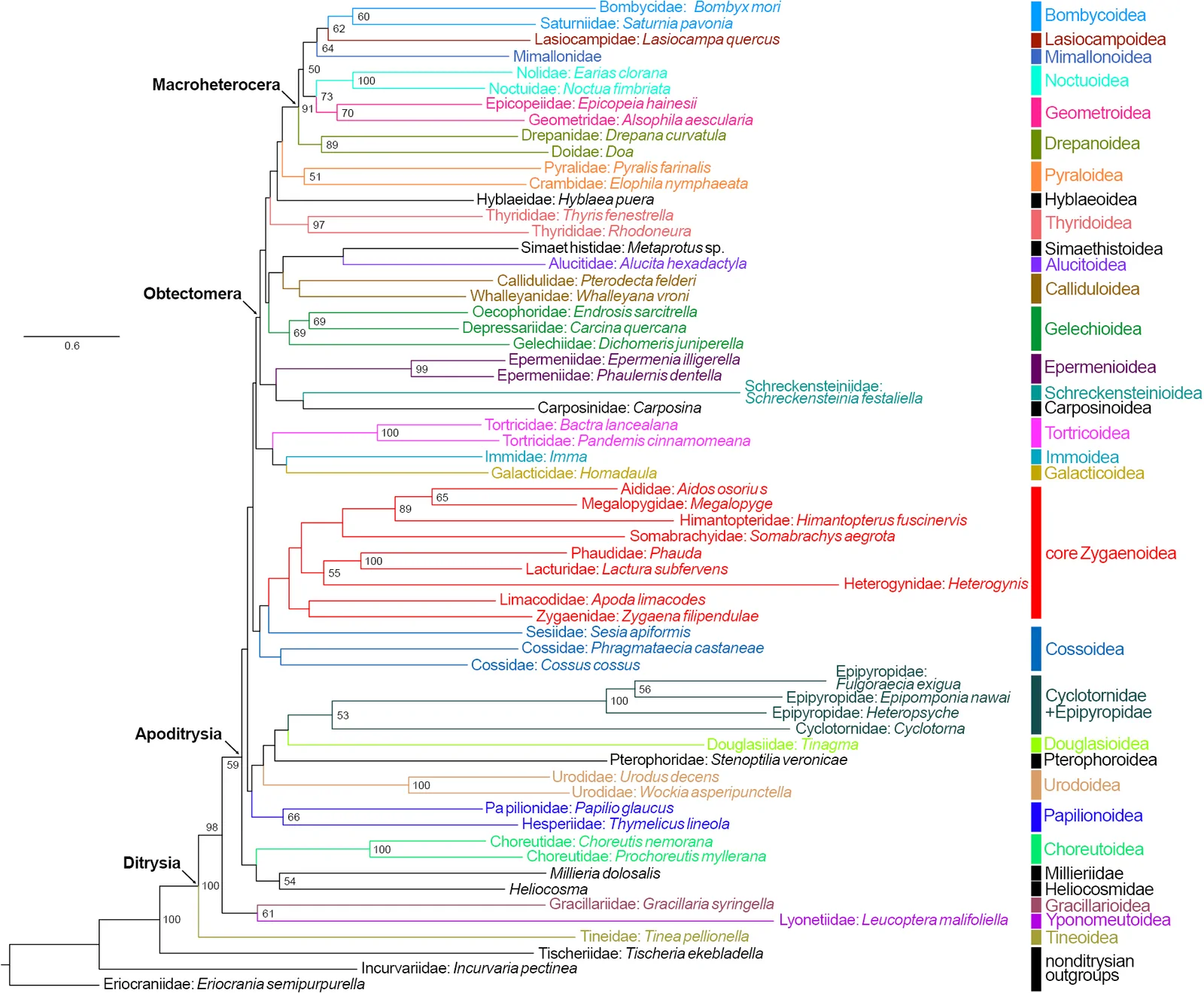
Maximum likelihood (ML) tree constructed with RAxML-NG for PCG123 dataset. Node labels indicate bootstrap support values (BS > 50 shown). Tip label format: full family and species names when all genes originate from the same species; family and genus names when genes come from different species of the same genus; family name only when genes are derived from different genera.

**Figure 6.**
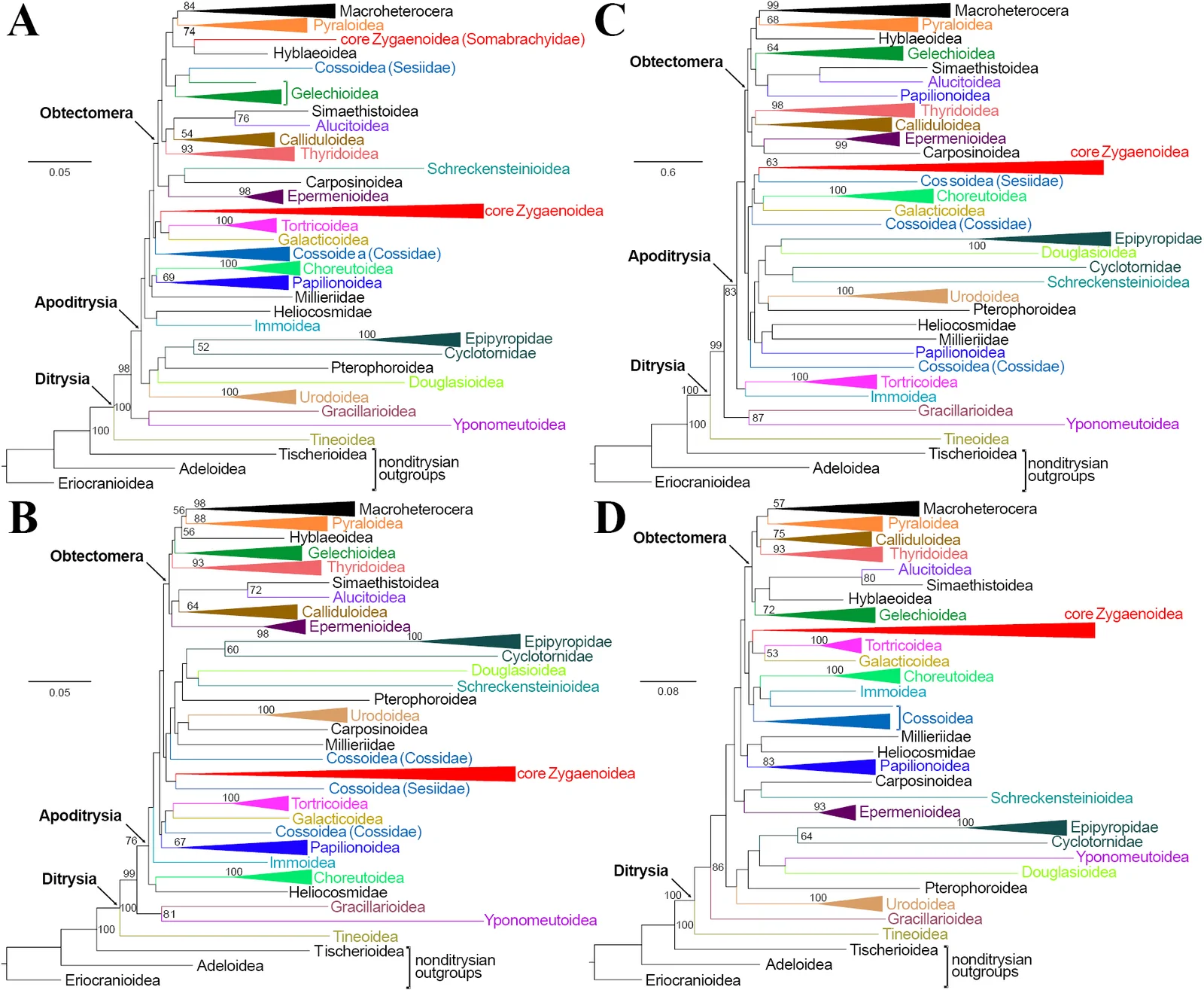
Partially collapsed Maximum likelihood (ML) trees constructed with RAxML-NG. Node labels indicate bootstrap support values (BS > 50 shown). **A–D**. ML trees for the PCG12, PCG12R, PCG123R, and AA datasets, respectively.

Different combinations of datasets, partitioning schemes, and tree-building methods generated 11 phylogenetic trees of Ditrysia (Figs 2, 3, 4, 5, 6). Among them, the AA Bayesian inference (BI) tree constructed using the site-heterogeneous substitution model (CAT+GTR) was selected as our primary tree (Fig. 2). This model has been shown to significantly improve model fit and reduce long-branch attraction (LBA) artifacts, making it more suitable for resolving deep and rapid phylogenetic radiations (e.g., Cai et al. 2023; Cai 2024), despite its higher computational demand. Our subsequent discussion is mainly based on the topology of this tree, with broad comparisons to the other trees (Figs 3, 4, 5, 6) generated using site-homogeneous models where necessary.

The topology of our primary tree of Ditrysia was largely congruent with several most recent lepidopteran phylogenomic studies (Mayer et al. 2021; Chen et al. 2025). Our results generally supported the division of Ditrysia into three nested clades: Apoditrysia, Obtectomera, and Macroheterocera, as summarized by Mitter et al. (2017).

At the base of Ditrysia, Gracillarioidea and Yponomeutoidea clustered together as a single clade and formed the sister group to the monophyletic Apoditrysia in almost all trees with strong support (PP: 0.99–0.1, BS: 98–99). This topology has been well supported by numerous previous studies (Breinholt et al. 2018; Kawahara et al. 2019; Mayer et al. 2021; Rota et al. 2022; Chen et al. 2025).

Our constituent superfamilies of Obtectomera were nearly identical to those of Mitter et al. (2017) and Chen et al. (2025), but differed in node-by-node topologies. Macroheterocera (consisting of large moths) was recovered as monophyletic within Obtectomera with moderate to strong nodal support (PP: 0.935–1, BS: 57–99) in all our trees. The clade (Macroheterocera + Pyraloidea) + Hyblaeoidea was recovered in half of our trees. However, Papilionoidea (butterflies) was recovered as sister to this clade only in our primary tree (PP = 0.78); in all other trees, it was placed outside Obtectomera with low nodal support. Calliduloidea and Thyridoidea were recovered as sister groups within Obtectomera in most of our trees, and their sibling relationship has been reinforced by multiple previous studies (Breinholt et al. 2018; Kawahara et al. 2019; Mayer et al. 2021; Rota et al. 2022). Carposinoidea, Epermenioidea, and Schreckensteinioidea tended to form an agglomeration, always with low nodal support, and were unstably positioned either within or outside Obtectomera across different trees. This agglomeration achieved its highest nodal support in our primary tree (PP = 0.66) as the sister group of Calliduloidea + Thyridoidea. The close relationship between Carposinoidea and Epermenioidea has been recovered in previous studies (Heikkilä et al. 2015; Chen et al. 2025); however, Schreckensteinioidea, which has historically been placed outside Obtectomera without a clear position, clustered with Carposinoidea (mostly accepted as an obtectomeran superfamily) in more than half of our trees, albeit the nodal support remained consistently low. Simaethistoidea, sampled for the first time in a phylogenetic study, consistently clustered together with Alucitoidea (many-plumed moths) within Obtectomera with high support (PP: 0.635–1, BS: 72–80). Although only a *COX1* barcode sequence was included for its representative, it is nonetheless noteworthy that Simaethistoidea may be a member of Obtectomera, a possibility that warrants further investigation.

Regarding the other apoditrysian superfamilies outside Obtectomera, the phylogenetic positions of two families, Heliocosmidae and Millieriidae, which could not be confidently assigned to any superfamily, remained unresolved in our analyses. However, unexpectedly, in half of our trees (though not in the primary tree), the two families clustered together into a monophyletic group, sometimes with very high nodal support (Fig. 3, PP = 1). The three representatives of Cossoidea (one from Sesiidae and two from Cossidae) were problematic, as they never formed a monophyletic group in any of our trees, consistent with findings from many previous studies (e.g., Bazinet et al. 2013; Kawahara et al. 2019; Mayer et al. 2021; Chen et al. 2025). Their positions were quite unstable, particularly that of Sesiidae. The relationship between Cossoidea and core Zygaenoidea could not be conclusively resolved. The observed pattern was that Cossidae and core Zygaenoidea were consistently recovered not far apart within a large clade that included varying other groups across different trees (though not in the primary tree). The position of Sesiidae varied even more. Nevertheless, core Zygaenoidea + Cossoidea did form a monophylum in one tree (Fig. 5), albeit with very low nodal support, and in two other trees with Galacticoidea nested within (Figs 3, 4C).

Zygaenoidea*s. l*., the focus of this study, never formed a monophylum in any of our trees. Cyclotornidae and Epipyropidae were recovered as sisters in most of our trees, with nodal support ranging from moderate to strong (PP: 0.5–1, BS: 52–64), except in one tree (Fig. 6C), where the two families were placed in the same clade but separated by two small superfamilies: Douglasioidea and Schreckensteinioidea. While our results are largely consistent with a sister relationship between these two ectoparasitic moth families, the support was not universal across all analyses, and the long branches leading to these lineages raise the possibility of long-branch attraction artifacts. These two families were almost always placed apart from core Zygaenoidea and Cossoidea, typically near the base of Apoditrysia. The sole exception occurred in the primary tree (Fig. 2), where they formed a clade with Douglasioidea and Cossidae, but with negligibly low nodal support. Our results consistently rejected a close relationship between Cyclotornidae + Epipyropidae and either core Zygaenoidea or Cossoidea. Instead, these two families formed a quite isolated group near the base of Apoditrysia, possibly with Douglasioidea as their closest relative. Based on our results, we support the exclusion of both Cyclotornidae and Epipyropidae from Zygaenoidea, regardless of whether they form a sister pair or two independent lineages.

The nine extant families of the core Zygaenoidea consistently formed a monophylum across all our trees, and support was particularly strong in the BI trees (PP: 0.729–0.998). The only exceptions were the BI and ML trees based on the PCG12 dataset (Figs 4A, 6A). In those trees, Somabrachyidae was placed outside the core Zygaenoidea, most likely because only one gene (*NAD1*) was included for this family in these analyses, leading to insufficient phylogenetic signals.

Our phylogenetic evidence supports the monophyly of Zygaenoidea (*sensu stricto*) composed of nine families, with Cyclotornidae and Epipyropidae falling outside this clade.

## Conclusions

Our phylogenetic analyses demonstrated that Zygaenoidea, as traditionally defined, was non-monophyletic. The two ectoparasitic families, Cyclotornidae and Epipyropidae, never clustered with the core Zygaenoidea but instead were placed near the base of Apoditrysia, possibly with Douglasioidea as their closest relative. They were recovered as sisters in most analyses, though with variable support; given the long branches of these taxa, this relationship requires further testing with additional data. Nevertheless, our results clearly support the exclusion of both Cyclotornidae and Epipyropidae from Zygaenoidea. In contrast, the remaining nine families consistently formed a well-supported monophylum, which we hereby propose as Zygaenoidea*s. str*. Our study also provides a refined phylogenetic framework for Ditrysia, and offers novel insights into the placements of several problematic lineages, including Simaethistoidea, Heliocosmidae, and Millieriidae. These findings resolve long-standing uncertainties regarding the ectoparasitic families and lay a solid foundation for future taxonomic and evolutionary studies of Zygaenoidea.

Several factors, however, should be considered when interpreting our results. Nodal support values were only moderate despite the large number of genes analyzed, largely because about two-thirds of the loci were mitochondrial. Unlike nuclear genes, which are commonly used for resolving higher-level relationships among Lepidoptera, mitochondrial sequences evolve rapidly and tend to reach saturation over time, which may weaken their ability to recover deep evolutionary nodes. This is particularly relevant for Cyclotornidae and Epipyropidae, both of which exhibited relatively long branches, a pattern often associated with accelerated substitution rates in ectoparasitic lineages. Long-branch attraction may therefore have contributed to the recovery of these two families as sisters in some of our analyses. Future phylogenomic studies incorporating denser taxon sampling and increased nuclear gene representation will be essential to further test this relationship and improve overall nodal support.

## Additional information

### Artificial Intelligence (AI) use

The authors accept full responsibility for the content of the manuscript, including the disclosure of any use of AI.

No AI tools were used in the preparation of this manuscript.

### Author ORCIDs

Y. Cai https://orcid.org/0000-0001-6554-3784

A. Yin https://orcid.org/0000-0001-7614-9969

### Data availability

Data presented in this study are openly available in the GenBank repository under accession numbers: PZ157773–PZ157776.

## Supplementary materials

Supplementary material 1 Detailed information of the 62 samples Data type xlsx Explanation note **table SS1**. Detailed information of the 62 samples analyzed in this study, including superfamily and family assignments, species sources for nuclear and mitochondrial genes (noting composite samples constructed from different species), GenBank accession numbers, and mitogenome assembly status. https://binary.pensoft.net/file/1770783This dataset is made available under the Open Database License (http://opendatacommons.org/licenses/odbl/1.0/). The Open Database License (ODbL) is a license agreement intended to allow users to freely share, modify, and use this Dataset while maintaining this same freedom for others, provided that the original source and author(s) are credited. Yanpeng Cai, Aihui Yin

Supplementary material 2 Primers used for PCR amplification, partitioning schemes and substitution models Data type pdf Explanation note **table S2**. Primers used for PCR amplification. **tables S3–S12**. Partitioning schemes and substitution models determined by ModelFinder for ML and BI analyses using site-homogeneous models. https://binary.pensoft.net/file/1770784This dataset is made available under the Open Database License (http://opendatacommons.org/licenses/odbl/1.0/). The Open Database License (ODbL) is a license agreement intended to allow users to freely share, modify, and use this Dataset while maintaining this same freedom for others, provided that the original source and author(s) are credited. Yanpeng Cai, Aihui Yin
