# Supplementary material for: Investigating the phylogenetic placement of ectoparasitic families Cyclotornidae and Epipyropidae: Multigene insights into Zygaenoidea systematics (Lepidoptera)
Source: Zookeys. 2026 Sep 14;1291:283–301. doi: 10.3897/zookeys.1291.204651 (PMC13594989; doi:10.3897/zookeys.1291.204651)
Supplement: Supplementary file 1 [file zookeys-1291-283_article-204651__-s002.pdf]

Table S2. Primers used for polymerase chain reaction amplification.

Gene region	Forward primer		Reverse primer		Reference
	Name	Sequence	Name	Sequence	
**EF-1α** elongation factor-1a	Starsky	5' CACATYAACATTGTCGTSATYGG 3'	Luck	5' CATRTTGTCKCCGTGCCAKCC 3'	Cho et al. 1995
	EF51.9	5' CARGACGTATACAAAATCGG 3'	EFrcM4	5' ACAGCVACKGTYTGYCTCATRTC 3'	Cho et al. 1995
**IDH** isocitrate dehydrogenase	IDHdeg27F	5' GGWGAYGARATGACNAGRATHATHTGG 3'	IDHdegR	5' TTYTTRCAIGCCCANACRAANCCNCC 3'	Wahlberg and Wheat 2008
**RpS5** ribosomal protein S5	RpS5F	5' ATGGCNGARGARAAYTGGAAYGA 3'	RpS5R	5' CGGTTRGAYTTRGCAACACG 3'	Wahlberg and Wheat 2008

Cho, S.; Mitchell, A.; Regier, J.C.; Mitter, C.; Poole, R.W.; Friedlander, T.P.; Zhao, S. A highly conserved nuclear gene for low-level phylogenetics: elongation factor-1α recovers morphology-based tree for heliothine moths. *Mol. Biol. Evol.* **1995**, 12, 650–656. https://doi.org/10.1093/oxfordjournals.molbev.a040245.

Wahlberg, N.; Wheat, C.W. Genomic outposts serve the phylogenomic pioneers: designing novel nuclear markers for genomic DNA extractions of Lepidoptera. *Syst. Biol.* **2008**, 57, 231–242. https://doi.org/10.1080/10635150802004406.

Table S3. Partitioning scheme and corresponding substitution models determined by ModelFinder for the PCG123R dataset in the ML analysis. The p1, p2 and p3 denote the first, second and third codon positions of each PCG, respectively.

Partitions	Models	Data partitions
P1	GTR+F+I+G	ATP6p1, COX2p1, COX3p1
P2	GTR+F+I+G	ATP6p2, COX3p2, CYTBp2, NAD2p2, NAD3p2
P3	GTR+F+I+G	ATP6p3, COX1p3, NAD3p3
P4	GTR+F+I+G	ATP8p1, NAD2p1, NAD3p1
P5	GTR+F+I+G	ATP8p2, NAD6p2
P6	GTR+F+G	ATP8p3, NAD2p3, NAD6p3
P7	GTR+F+I+G	CADp1
P8	GTR+F+I+G	CADp2, COX2p2, MDHp2, WINGLESSp2
P9	GTR+F+I+G	CADp3
P10	GTR+F+I+G	COX1p1
P11	GTR+F+I+G	COX1p2, EF1Ap2, GAPDHp2, IDHp2
P12	GTR+F+G	COX2p3, CYTBp3
P13	GTR+F+G	COX3p3
P14	GTR+F+I+G	CYTBp1
P15	GTR+F+I+G	EF1Ap1, GAPDHp1, MDHp1, RPS5p1
P16	GTR+I+G	DNA, EF1Ap3, GAPDHp3, RPS5p3
P17	GTR+I+G	DNA, IDHp1, WINGLESSp1
P18	GTR+F+I+G	IDHp3
P19	GTR+F+I+G	MDHp3
P20	GTR+F+I+G	NAD1p1, NAD4Lp1, NAD4p1, NAD5p1
P21	GTR+F+I+G	NAD1p2, NAD4Lp2, NAD4p2, NAD5p2
P22	GTR+F+G	NAD1p3, NAD4Lp3, NAD4p3
P23	GTR+F+G	NAD5p3
P24	GTR+F+G	NAD6p1
P25	GTR+I+G	DNA, RPS5p2
P26	GTR+F+I+G	WINGLESSp3
P27	GTR+F+I+G	*rrnL*
P28	GTR+F+I+G	*rrnS*

Table S4. The partitioning scheme and corresponding substitution models determined by ModelFinder for the PCG123R dataset in the BI analysis. The p1, p2 and p3 denote the first, second and third codon positions of each PCG, respectively.

Partitions	Models	Data partitions
P1	GTR+F+I+G4	ATP6p1, COX2p1, COX3p1, CYTBp1
P2	GTR+F+I+G4	ATP6p2, COX3p2, CYTBp2, NAD2p2, NAD3p2
P3	GTR+F+I+G4	ATP6p3, COX1p3, NAD3p3
P4	GTR+F+I+G4	ATP8p1, ATP8p2, NAD2p1, NAD3p1
P5	HKY+F+I+G4	ATP8p3, COX3p3
P6	GTR+F+I+G4	CADp1, IDHp1
P7	GTR+F+I+G4	CADp2, COX2p2, MDHp2, WINGLESSp2
P8	GTR+F+I+G4	CADp3
P9	GTR+F+I+G4	COX1p1
P10	GTR+F+I+G4	COX1p2, EF1Ap2, GAPDHp2, IDHp2
P11	GTR+F+G4	COX2p3, CYTBp3
P12	GTR+F+I+G4	EF1Ap1, GAPDHp1, MDHp1, RPS5p1
P13	GTR+I+G4	EF1Ap3, GAPDHp3, RPS5p3
P14	GTR+F+I+G4	IDHp3
P15	GTR+F+I+G4	MDHp3
P16	GTR+F+I+G4	NAD1p1, NAD4Lp1, NAD4p1, NAD5p1
P17	GTR+F+I+G4	NAD1p2, NAD4Lp2, NAD4p2, NAD5p2
P18	GTR+F+I+G4	NAD1p3, NAD4p3
P19	GTR+F+G4	NAD2p3, NAD6p3
P20	HKY+F+G4	NAD4Lp3
P21	GTR+F+G4	NAD5p3
P22	GTR+F+G4	NAD6p1
P23	GTR+F+G4	NAD6p2
P24	SYM+I+G4	RPS5p2
P25	GTR+F+I+G4	*rrnL*, *rrnS*
P26	GTR+I+G4	WINGLESSp1
P27	GTR+F+I+G4	WINGLESSp3

Table S5. The partitioning scheme and corresponding substitution models determined by ModelFinder for the PCG123 dataset in the ML analysis. The p1, p2 and p3 denote the first, second and third codon positions of each PCG, respectively.

**Partitions**	**Models**	**Data partitions**
P1	GTR+F+I+G	ATP6p1, COX2p1, COX3p1
P2	GTR+F+I+G	ATP6p2, COX3p2, CYTBp2, NAD2p2, NAD3p2
P3	GTR+F+I+G	ATP6p3, COX1p3
P4	GTR+F+I+G	ATP8p1, NAD2p1, NAD3p1
P5	GTR+F+G	ATP8p2, NAD6p2
P6	GTR+F+I+G	ATP8p3, COX3p3
P7	GTR+F+I+G	CADp1, IDHp1
P8	GTR+F+I+G	CADp2, COX2p2
P9	GTR+F+I+G	CADp3
P10	GTR+F+I+G	COX1p1
P11	GTR+F+I+G	COX1p2, EF1Ap2, GAPDHp2, IDHp2
P12	GTR+F+G	COX2p3, CYTBp3
P13	GTR+F+I+G	CYTBp1
P14	GTR+F+I+G	EF1Ap1, GAPDHp1, MDHp1, RPS5p1
P15	GTR+I+G	EF1Ap3, GAPDHp3, RPS5p3
P16	GTR+F+I+G	IDHp3
P17	GTR+I+G	MDHp2, WINGLESSp2
P18	GTR+F+I+G	MDHp3
P19	GTR+F+I+G	NAD1p1, NAD4Lp1, NAD4p1, NAD5p1
P20	GTR+F+I+G	NAD1p2, NAD4Lp2, NAD4p2, NAD5p2
P21	GTR+F+I+G	NAD1p3, NAD4p3
P22	GTR+F+G	NAD2p3
P23	GTR+F+I+G	NAD3p3, NAD6p3
P24	GTR+F+G	NAD4Lp3
P25	GTR+F+G	NAD5p3
P26	GTR+F+G	NAD6p1
P27	GTR+I+G	RPS5p2
P28	GTR+I+G	WINGLESSp1
P29	GTR+F+I+G	WINGLESSp3

Table S6. The partitioning scheme and corresponding substitution models determined by ModelFinder for the PCG123 dataset in the BI analysis. The p1, p2 and p3 denote the first, second and third codon positions of each PCG, respectively.

Partitions	Models	Data partitions
P1	GTR+F+I+G4	ATP6p1, COX3p1, CYTBp1
P2	GTR+F+I+G4	ATP6p2, COX3p2, CYTBp2, NAD2p2, NAD3p2
P3	GTR+F+I+G4	ATP6p3, COX1p3, NAD3p3
P4	GTR+F+I+G4	ATP8p1, ATP8p2, NAD2p1, NAD3p1
P5	GTR+F+G4	ATP8p3, NAD6p1
P6	GTR+F+I+G4	CADp1, IDHp1
P7	GTR+F+I+G4	CADp2, COX2p2, MDHp2, WINGLESSp2
P8	GTR+F+I+G4	CADp3
P9	GTR+F+I+G4	COX1p1, COX2p1
P10	GTR+F+I+G4	COX1p2
P11	GTR+F+I+G4	COX2p3, CYTBp3
P12	HKY+F+I+G4	COX3p3
P13	GTR+F+I+G4	EF1Ap1, GAPDHp1, MDHp1, RPS5p1
P14	GTR+F+I+G4	EF1Ap2, GAPDHp2, IDHp2
P15	GTR+I+G4	EF1Ap3, GAPDHp3, RPS5p3
P16	GTR+F+I+G4	IDHp3, MDHp3
P17	GTR+F+I+G4	NAD1p1, NAD4Lp1, NAD4p1, NAD5p1
P18	GTR+F+I+G4	NAD1p2, NAD4Lp2, NAD4p2, NAD5p2
P19	GTR+F+I+G4	NAD1p3, NAD4p3, NAD5p3
P20	GTR+F+G4	NAD2p3, NAD6p3
P21	HKY+F+G4	NAD4Lp3
P22	GTR+F+G4	NAD6p2
P23	GTR+I+G4	RPS5p2
P24	GTR+I+G4	WINGLESSp1
P25	GTR+F+I+G4	WINGLESSp3

Table S7. The partitioning scheme and corresponding substitution models determined by ModelFinder for the PCG12R dataset in the ML analysis. The p1 and p2 denote the first and second codon positions of each PCG, respectively.

**Partitions**	**Models**	**Data partitions**
P1	GTR+F+I+G	ATP6p1, NAD2p1, NAD3p1
P2	GTR+F+I+G	ATP6p2, COX2p2, COX3p2, CYTBp2
P3	GTR+F+I+G	ATP8p1
P4	GTR+F+I+G	ATP8p2, NAD6p2
P5	GTR+F+I+G	CADp1
P6	GTR+F+I+G	CADp2
P7	GTR+F+I+G	COX1p1
P8	GTR+F+I+G	COX1p2
P9	GTR+F+I+G	COX2p1
P10	GTR+F+I+G	COX3p1
P11	GTR+F+I+G	CYTBp1
P12	GTR+F+I+G	EF1Ap1, GAPDHp1, RPS5p1
P13	GTR+F+I+G	EF1Ap2, GAPDHp2
P14	GTR+I+G	IDHp1, WINGLESSp1
P15	GTR+F+I+G	IDHp2, MDHp2, WINGLESSp2
P16	GTR+F+I+G	MDHp1
P17	GTR+F+I+G	NAD1p1, NAD4Lp1, NAD4p1, NAD5p1
P18	GTR+F+I+G	NAD1p2, NAD4p2, NAD5p2
P19	GTR+F+I+G	NAD2p2, NAD3p2, NAD4Lp2
P20	GTR+F+G	NAD6p1
P21	GTR+I+G	RPS5p2
P22	GTR+F+I+G	*rrnL*
P23	GTR+F+I+G	*rrnS*

Table S8. The partitioning scheme and corresponding substitution models determined by ModelFinder for the PCG12R dataset in the BI analysis. The p1 and p2 denote the first and second codon positions of each PCG, respectively.

Partitions	Models	Data partitions
P1	GTR+F+I+G4	ATP6p1, COX3p1
P2	GTR+F+I+G4	ATP6p2, CADp2, COX2p2, COX3p2, CYTBp2
P3	GTR+F+I+G4	ATP8p1, ATP8p2, NAD2p1, NAD3p1
P4	GTR+F+I+G4	CADp1
P5	GTR+F+I+G4	COX1p1, COX2p1
P6	GTR+F+I+G4	COX1p2, EF1Ap2, GAPDHp2, IDHp2
P7	GTR+F+I+G4	CYTBp1
P8	GTR+F+I+G4	EF1Ap1, GAPDHp1, MDHp1, RPS5p1, WINGLESSp2
P9	GTR+I+G4	IDHp1, WINGLESSp1
P10	GTR+I+G4	MDHp2
P11	GTR+F+I+G4	NAD1p1, NAD4Lp1, NAD4p1, NAD5p1
P12	GTR+F+I+G4	NAD1p2, NAD4Lp2, NAD4p2, NAD5p2
P13	GTR+F+I+G4	NAD2p2, NAD3p2
P14	GTR+F+G4	NAD6p1
P15	GTR+F+I+G4	NAD6p2
P16	SYM+I+G4	RPS5p2
P17	GTR+F+I+G4	*rrnL*
P18	GTR+F+I+G4	*rrnS*

Table S9. The partitioning scheme and corresponding substitution models determined by ModelFinder for the PCG12 dataset in the ML analysis. The p1 and p2 denote the first and second codon positions of each PCG, respectively.

Partitions	Models	Data partitions
P1	GTR+F+I+G	ATP6p1, COX2p1, COX3p1
P2	GTR+F+I+G	ATP6p2, CADp2, COX3p2, CYTBp2
P3	GTR+F+I+G	ATP8p1, ATP8p2, NAD2p1, NAD3p1
P4	GTR+F+I+G	CADp1
P5	GTR+F+I+G	COX1p1
P6	GTR+F+I+G	COX1p2, COX2p2, EF1Ap2, GAPDHp2, IDHp2, MDHp2, RPS5p2
P7	GTR+F+I+G	CYTBp1
P8	GTR+F+I+G	EF1Ap1, GAPDHp1
P9	GTR+F+I+G	IDHp1
P10	GTR+F+I+G	MDHp1, RPS5p1, WINGLESSp2
P11	GTR+F+I+G	NAD1p1, NAD4Lp1, NAD4p1, NAD5p1
P12	GTR+F+I+G	NAD1p2, NAD4Lp2, NAD4p2, NAD5p2
P13	GTR+F+I+G	NAD2p2, NAD3p2
P14	GTR+F+G	NAD6p1
P15	GTR+F+I+G	NAD6p2
P16	GTR+I+G	WINGLESSp1

Table S10. The partitioning scheme and corresponding substitution models determined by ModelFinder for the PCG12 dataset in the BI analysis. The p1 and p2 denote the first and second codon positions of each PCG, respectively.

Partitions	Models	Data partitions
P1	GTR+F+I+G4	ATP6p1, COX2p1, COX3p1
P2	GTR+F+I+G4	ATP6p2, COX2p2, COX3p2, CYTBp2
P3	GTR+F+I+G4	ATP8p1, ATP8p2, NAD2p1, NAD3p1
P4	GTR+F+I+G4	CADp1
P5	GTR+F+I+G4	CADp2
P6	GTR+F+I+G4	COX1p1, NAD3p2
P7	GTR+F+I+G4	COX1p2
P8	GTR+F+I+G4	CYTBp1
P9	GTR+F+I+G4	EF1Ap1, GAPDHp1, RPS5p1
P10	GTR+F+I+G4	EF1Ap2, GAPDHp2, MDHp2
P11	GTR+F+I+G4	IDHp1
P12	GTR+F+I+G4	IDHp2
P13	GTR+F+I+G4	MDHp1, WINGLESSp2
P14	GTR+F+I+G4	NAD1p1, NAD5p1
P15	GTR+F+I+G4	NAD1p2, NAD4Lp2, NAD4p2, NAD5p2
P16	GTR+F+I+G4	NAD2p2
P17	GTR+F+I+G4	NAD4Lp1, NAD4p1
P18	GTR+F+G4	NAD6p1
P19	GTR+F+I+G4	NAD6p2
P20	SYM+I+G4	RPS5p2
P21	GTR+I+G4	WINGLESSp1

Table S11. The partitioning scheme and corresponding substitution models determined by ModelFinder for the AA dataset in the ML analysis.

Partitions	Models	Data partitions
P1	MTART+F+I+G	ATP6, ATP8, NAD2, NAD3
P2	LG+I+G	CAD
P3	LG+F+I+G	COX1
P4	MTART+I+G	COX2
P5	MTART+F+I+G	COX3, CYTB
P6	JTTDCMUT+I+G	EF1A, GAPDH, RPS5
P7	LG+I+G	IDH, MDH
P8	MTART+F+I+G	NAD1, NAD4, NAD5
P9	MTART+F+I+G	NAD4L, NAD6
P10	LG+I+G	WINGLESS

Table S12. The partitioning scheme and corresponding substitution models determined by ModelFinder for the AA dataset in the BI analysis.

**Partitions**	**Models**	**Data partitions**
P1	MTREV+F+I+G4	ATP6, ATP8, NAD2, NAD3, NAD4L
P2	JTT+I+G4	CAD
P3	MTREV+F+G4	COX1, COX2
P4	MTREV+F+G4	COX3, CYTB
P5	DAYHOFF+I+G4	EF1A, GAPDH, RPS5
P6	JTT+I+G4	IDH, MDH
P7	MTREV+F+I+G4	NAD1, NAD4, NAD5
P8	MTREV+F+G4	NAD6
P9	JTT+I+G4	WINGLESS
